# Molecular Characterization of an H3N2 Canine Influenza Virus Isolated from a Dog in Jiangsu, China, in 2025

**DOI:** 10.3390/vetsci13010032

**Published:** 2025-12-29

**Authors:** Jingwen Peng, Xinyu Miao, Xinyi Zhang, Zhifan Li, Yiling Wang, Guofang Liu, Lei Na, Nuo Xu, Daxin Peng

**Affiliations:** 1 Department of Veterinary Clinical Sciences, College of Veterinary Medicine, Nanjing Agricultural University, Nanjing 210095, China; jingwenp@njau.edu.cn (J.P.);; 2 Nanjing Agricultural University Veterinary Teaching Hospital, Nanjing 210095, China; 3 College of Veterinary Medicine, Yangzhou University, Yangzhou 225009, China; 4 The Department of Animal Husbandry and Veterinary Medicine, Jiangsu Vocational College of Agriculture and Forestry, Jurong 212400, China

**Keywords:** H3N2 influenza virus, canine influenza, antigenic sites, hemagglutinin, glycosylation

## Abstract

To investigate the molecular characteristics of H3N2 canine influenza viruses circulating in Jiangsu, China, we isolated a H3N2 strain (A/Canine/Nanjing/CnNj01-2025) from a dog presenting with respiratory signs at the Veterinary Teaching Hospital of Nanjing Agricultural University. All eight gene segments were sequenced and compared with those of two human H3N2 strains and five avian H3N2 strains. Antigenicity and receptor-binding properties were also assessed. Phylogenetic analysis revealed that the canine isolate descended from the avian lineage and formed an independent evolutionary clade, while the human strains were more distantly related to the avian lineage. Glycosylation analysis of the HA protein revealed that the canine strain carried seven N-glycosylation sites, including a unique site at residue 97/81 (HA/H3 numbering), which serves as a molecular signature of the canine strain. Several amino-acid substitutions were identified in major antigenic sites, including D97/81N, A176/160T, N204/188D, V212/196I, and W237/222L. Analysis of internal genes showed that the canine strain harbored PB2 292T and 590S mammalian adaptation mutations, which are also present in human strains. Hemagglutination inhibition (HI) assays of the canine strain indicated moderate serologic cross-reactivity with a human H3N2 antiserum (16-fold reduction), whereas avian strains showed no cross-reactivity. Receptor-binding assays demonstrated that the virus retained predominant α-2,3 sialic acid binding, comparable to that of avian influenza viruses, and gained a modest affinity for human-type α-2,6 sialic acid receptors. Therefore, the canine H3N2 virus has undergone significant antigenic drift, developed partial serological cross-reactivity with human strains, and acquired detectable but limited binding affinity for human-type receptors. Overall, our findings suggest that the current canine H3N2 influenza virus exhibits distinct genetic and antigenic variations from human and avian strains. Continuous molecular and serological surveillance of canine influenza viruses is therefore warranted to monitor their evolutionary trends and assess the potential for cross-species transmission.

## Simple Summary

Canine influenza virus H3N2, which originally derived from avian strains, continues to evolve in dogs. In this study, we isolated an H3N2 virus from a dog in Jiangsu, China, and analyzed its antigenic and molecular characteristics. Hemagglutination inhibition assays using human antiserum indicated partial cross-reactivity with the canine virus, while no reactivity was detected with avian strains. Five amino acid substitutions and altered glycosylation patterns contributed to distinct antigenic properties in comparison with human and avian H3N2 viruses. Receptor-binding assays revealed strong affinity for avian-type a-2,3 receptors and limited binding to human-type a-2,6 receptors. These findings highlight the different genetic and antigenic characteristics of canine H3N2 virus from human and avian strains, and the necessity for sustained surveillance of canine H3N2 virus in canine populations.

## 1. Introduction

Canine influenza virus (CIV) H3N2 has circulated continuously in dog populations since its emergence in Asia around 2004 and has become one of the predominant influenza A subtypes infecting canines [1,2,3]. Early genomic analyses revealed that all eight gene segments of H3N2 CIV originated from avian influenza viruses, indicating a complete cross-species transmission event followed by successful establishment and sustained circulation in dogs [4,5]. Subsequent phylogenetic analysis showed that, during the early phase of spread in Asia, H3N2 CIV rapidly diversified into several geographically distinct lineages, with relatively independent transmission patterns emerging in China and South Korea [5]. In 2015, H3N2 CIV was first introduced into North America, resulting in a large outbreak in Chicago and surrounding regions. Additional introductions in 2017 and 2018 represented independent importation events originating from distinct Asian sources [6,7]. A recent comprehensive phylogenetic analysis of 297 global CIV-H3N2 whole-genome sequences identified six distinct evolutionary clades (Clades 1–6), each associated with specific geographic dissemination and epidemiological events. For example, Clade 1 and Clade 2 represent geographically distinct lineages that were established in China and South Korea, respectively. The Clade 3 virus that emerged in the United States is derived from a Clade 2 virus. Clade 4 contains the secondary outbreaks of H3N2 CIV in the United States and Canada. Clade 5 includes isolates from 2018–2019 in China, the United States, and Canada. Clade 6 contains only recent isolates (2021–2023) from the United States [1]. The stepwise accumulation of mutations across these clades reflects ongoing adaptive evolution of the virus in canine hosts. Surveillance studies in China and South Korea further corroborated this dynamic evolutionary process, revealing region-specific lineages and elevated nonsynonymous-to-synonymous substitution ratios (dN/dS) across all gene segments, consistent with host-associated selective pressures [8,9,10]. Moreover, mounting evidence suggests that H3N2 canine influenza viruses have undergone strong immune-driven and host-adaptive evolution during sustained circulation in dogs, with positive selection acting on key antigenic and host-interactive regions of the HA and NA genes, ultimately promoting independent evolutionary trajectories between Chinese and Korean lineages [4,11].

Although H3N2 CIV initially exhibited limited transmission efficiency among dogs, gradual enhancement of transmission ability and ongoing antigenic evolution have facilitated its widespread circulation in canine populations [9]. Importantly, H3N2 CIV has demonstrated the capacity for cross-species transmission. Natural infections have been reported in cats both in South Korea and the USA, and serological evidence suggests exposure in horses with close contact to infected dogs [12,13,14]. More recently, a novel H3N2 canine/human reassortant virus was isolated from zoo-housed golden monkeys, where it was associated with fatal disease, highlighting the broader host range and reassortment potential of H3N2 CIV [15]. Through step-by-step evolution, H3N2 CIV has progressively acquired human-adaptive features in surface and internal genes and now circulates endemically in dog populations, which raises theoretical concerns regarding human infection [4,16]. However, no confirmed cases of natural human infections with H3N2 CIV have been reported to date, and formal spillover risk assessments have concluded that the current risk to the human populations remains low [17].

Despite nearly two decades of circulation in dogs, relatively few studies have systematically compared the molecular characteristics, antigenicity, and receptor-binding properties of CIV-H3N2 with those of human and avian H3N2 viruses. Such comparative analysis is essential for understanding host adaptation, antigenic evolution, and the mechanisms underlying cross-species transmission. In the present study, we isolated a canine H3N2 virus from a veterinary hospital in China and performed comprehensive characterization using whole-genome sequencing, phylogenetic analysis, glycosylation site prediction, antigenic site comparison, serological cross-reactivity testing, and receptor-binding assays. By placing the canine isolate in the context of human and avian H3N2 lineages, this study provides novel insights into the evolutionary trajectory, host adaptation, and potential public health relevance of CIV-H3N2.

## 2. Materials and Methods

### 2.1. Ethics Statement

Animal care, housing, feeding, sampling, observation, and environmental conditions complied with the experimental animal welfare and ethics guidelines of the Jiangsu Administrative Committee for Laboratory Animals (Permission number: SYXK-SU-2021-0027). All procedures involving animals were conducted in accordance with institutional ethical guidelines.

### 2.2. Virus Isolation, Identification, and Genomic Sequences

A nasal and oropharyngeal swab sample was collected from a domestic dog presenting with respiratory signs at the Veterinary Teaching Hospital of Nanjing Agricultural University, Nanjing, Jiangsu Province, China, in 2025. The sample was inoculated into the allantoic cavity of 10-day-old specific-pathogen-free (SPF) embryonated chicken eggs and incubated at 37 °C for 72 h. Following incubation, the allantoic fluid was harvested, and a hemagglutination assay was performed using 1% guinea pig red blood cells to detect the presence of hemagglutination activity. Viral RNA was extracted from the positive allantoic fluid using a commercial viral RNA extraction kit (Tiangen Biotech Co., Ltd., Beijing, China). Reverse transcription was conducted using the HiScript IV RT SuperMix for qPCR (+gDNA wiper) kit (Vazyme Biotech Co., Ltd., Nanjing, China; Cat. No. R423-01), according to the manufacturer’s protocol. Briefly, the reaction mixture was incubated at 37 °C for 15 min for cDNA synthesis, followed by enzyme inactivation at 85 °C for 5 s. Influenza A virus infection was confirmed by RT-PCR amplification of the M gene [18]. After confirmation, all eight gene segments were amplified using universal primers specific to influenza A virus, as previously described by Hoffmann et al. [19]. The PCR products were sequenced by Tsingke Biotechnology Co., Ltd. (Beijing, China) using both Sanger sequencing and next-generation sequencing (NGS) platforms. The sequence data from both methods were integrated and analyzed to generate the complete genome sequence of the isolate.

### 2.3. Phylogenetic Analysis

A total of 104 representative H3N2 virus HA gene sequences were downloaded from the GISAID or GenBank database for phylogenetic analysis. Detailed information on the selected strains is provided in Supplementary Table S1. All sequences were first aligned using the MAFFT (v7.505) algorithm within the PhyloSuite platform (v1.2.2) under default parameters. The alignments were further manually inspected and corrected for mismatches to ensure high alignment quality. ModelFinder was then used to evaluate a range of nucleotide substitution models, and the best-fitting model was selected based on the Bayesian Information Criterion (BIC).

The phylogenetic tree was constructed using IQ-TREE software (v3.0.1) with the following settings: sequence type was set to auto-detect, the codon table was set to the standard genetic code, and the substitution model was set to Auto. Branch support was assessed using the Ultrafast Bootstrap method with 5000 replicates, and the SH-aLRT branch test with 1000 replicates was also enabled to enhance statistical robustness. Thread usage was automatically optimized, and the FreeRate model was applied for rate heterogeneity correction, with the minimum number of rate categories set to 2 and the maximum to 10. To ensure the stability of the results, the maximum number of iterations was set to 1000 and the minimum correlation coefficient was set to 0.90. The final phylogenetic tree was visualized and annotated using the Interactive Tree of Life (iTOL) (v7.3) online tool (https://itol.embl.de/, accessed on 20 November 2025).

### 2.4. Antigen Site Analysis

For HA protein antigenic site analysis, amino acid sequences corresponding to the five major antigenic sites (Sites A–E) were extracted from the alignment according to established H3 numbering conventions. Multiple sequence alignments were visualized using Jalview v2.11.3.2, with amino acids color-coded according to the Clustal X scheme to highlight biochemical properties and conservation patterns. Residues in antigenic site figures were labeled with dual numbering (HA position/H3 position) to facilitate comparison with published data.

### 2.5. Glycosylation Site Prediction and Structure Analysis

A total of 685 avian-derived, 349 canine-derived, and 19,527 human-derived H3N2 HA gene sequences (collected from January to August 2025) were retrieved from the GISAID database for glycosylation site analysis (Supplementary Table S2). Glycosylation sites were predicted based on the presence of the consensus N-X-S/T motif (where X represents any amino acid except proline). Strains with incomplete sequence information at corresponding sites were excluded from analysis. The percentage of glycosylation at each HA site (H3 numbering aligned with site equivalence) is shown for each host species. HA protein glycosylation sites were predicted using the NetNGlyc 1.0 online tool (https://services.healthtech.dtu.dk/services/NetNGlyc-1.0/, accessed on 1 November 2025). The three-dimensional structures of the HA proteins were generated via the AlphaFold online server (https://alphafoldserver.com/, accessed on 6 November 2025), and glycosylation modifications were added using the Glycoprotein Builder module from Glycam (https://glycam.org/, accessed on 10 November 2025). The final structural visualization and comparative analysis were performed in PyMOL (v2.4.0a0 Open-source).

### 2.6. Functional Analysis of Key Residues in Genomic Segments

Amino acid sequences of seven gene segments from the canine isolate A/Canine/Nanjing/CnNj01-2025, one human H3N2 strain, and five avian H3N2 strains (Table 1) were aligned using the MAFFT algorithm within the PhyloSuite platform under default parameters. Alignments were manually inspected and adjusted to ensure accuracy. Functionally important residues associated with mammalian adaptation, virulence, and receptor binding were identified and compared among strains based on published literature.

### 2.7. Hemagglutination Inhibition Assay

To prepare antiserum against the canine H3N2 strain, four-week-old specific pathogen-free (SPF) chickens were inoculated intranasally with 10^6.0^ EID_50_ of the virus in a total volume of 200 μL phosphate-buffered saline (PBS), administered twice at a 7-day interval. Whole blood was collected from the chickens at 21 days after the final inoculation, and serum was subsequently separated by centrifugation. The resulting antiserum was titrated, aliquoted, and stored at −80 °C for further use. Additionally, hemagglutination inhibition (HI) antigen and goat serum against the A/Sichuan/Jianyang/35/2023 were provided by the Jiangsu Center for Disease Control and Prevention, China.

The HI assay was performed in 96-well V-bottom microtiter plates using a two-fold serial dilution method. Serum samples were initially diluted at 1:10, with a final dilution of 1:1280, and 25 μL of each dilution was added per well. Virus antigens were standardized to 4 hemagglutination units and mixed with the diluted sera at 25 μL per well. After gentle mixing, the plate was incubated at room temperature for 30 min to allow antigen-antibody binding. Following incubation, 50 μL of 1% guinea pig red blood cell suspension was added to each well, the plate was gently mixed again, and allowed to stand at room temperature for 30 to 45 min. The inhibition of hemagglutination was then observed and recorded.

### 2.8. Receptor Binding Assay

To evaluate the receptor binding characteristic of the canine-derived isolate, receptor binding specificity was analyzed via a solid-phase binding assay with two different glycopolymers: an α-2,3-sialylglycopolymer (Neu5Acα2-3Galβ1-4GlcNAcβ1-pAP (para-aminophenyl)-alpha-polyglutamic acid (α-PGA)) (α-2,3-glycans) and an α-2,6-sialylglycopolymer (Neu5Acα2-6Galβ1-4GlcNAcβ1-pAP (para-aminophenyl)-alpha-polyglutamic acid (α-PGA)) (α-2,6-glycans), as previously described [20]. A/California/04/2009(H1N1), which only binds to α-2,6-glycans, and A/Mallard/Huadong/S/2005(H5N1), which only binds to α-2,3-glycans, were selected as control viruses. Two different glycan analogs were serially diluted in PBS and added to 96-well streptavidin-coated microtiter plates. The plates were blocked overnight with 5% BSA and subsequently incubated with 6 log_2_ hemagglutination titers of each virus per well overnight. Primary antibody consisted of chicken antiserum against A/Mallard/Huadong/S/2005 (H5N1) or the canine-derived isolate and mouse antiserum against A/California/04/2009 (H1N1) [21]. Enzyme-labeled goat antiserum against mice or chicken was used as the secondary antibody. Following the washing steps, the tetramethylbenzidine substrate solution was added successively. After reacting for 10 min, the reaction was stopped with 2 M H_2_SO_4_, and the absorbance at 450 nm was read for curve plotting.

### 2.9. Statistical Analysis

GraphPad Prism version 8 software was used to generate the graphs.

## 3. Results

### 3.1. Isolation and Molecular Characterization of Canine H3N2 Influenza Virus

In March 2025, a nasal and oropharyngeal swab sample was collected from a dog presenting with coughing, nasal discharge, and mild fever. Following inoculation into SPF embryonated chicken eggs, the hemagglutination assay revealed an HA titer of 6 log_2_ in the allantoic fluid, and RT-PCR analysis confirmed the presence of influenza A virus. Subsequent sequencing of all eight gene segments yielded complete coding sequences consistent with an H3N2 influenza virus with no evidence of coinfection with other subtypes. The canine H3N2 virus was designated as A/Canine/Nanjing/CnNj01-2025 (C1). The complete genomic sequences have been deposited in the GenBank database under accession numbers PX474839 (PB2), PX474838 (PB1), PX474837 (PA), PX474832 (HA), PX474835 (NP), PX474834 (NA), PX474833 (M), and PX474836 (NS). All viral strains and sequence information used in this study are listed in Table 1.

### 3.2. Phylogenetic Analysis of H3N2 Influenza Viruses Based on HA Gene Sequences

**Figure 1:**
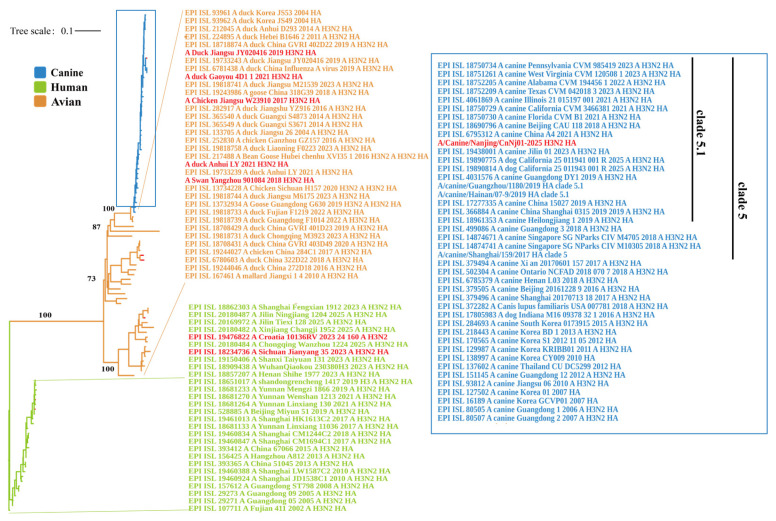
Phylogenetic analysis of HA genes within H3N2 influenza viruses. The maximum likelihood phylogenetic tree was constructed using IQ-TREE (v3.0.1) with the best-fit substitution model GTR+F+I+G4, as determined by ModelFinder, using 5000 ultrafast bootstrap replicates and 1000 SH-aLRT branch tests. The tree includes 104 representative H3N2 virus strains from GISAID database and strains from this study. The tree was rooted with EPI_ISL_107711/A/Fujian 411/2002/A_H3N2. Strains are color-coded by host origin: blue for canine isolates, green for human isolates, and orange for avian isolates. Scale bar indicates nucleotide substitutions per site (0.1). Bootstrap values ≥ 70% and SH-aLRT support values ≥ 80% are shown at key nodes. Strains from this study are highlighted in red.

A maximum-likelihood phylogenetic tree was constructed using the HA gene sequences of 104 representative H3N2 influenza virus strains. The resulting tree revealed clearly separated evolutionary branches corresponding to different host origins. Overall, three major clades were identified: human-derived, canine-derived, and avian-derived lineages. The human-derived viruses formed a distinct monophyletic cluster, within which both the WHO-reference vaccine strain A/Croatia/l0136RV/2023 and a human-derived strain A/Sichuan/Jianyang/35/2023 were located (Figure 1). The avian-derived viruses were located on the left and lower central regions of the tree. The five avian strains analyzed in this study: A/Chicken/Jiangsu/W23910/2017, A/Duck/Jiangsu/JY020416/2019, A/Swan/Yangzhou/901084/2018, A/Duck/Anhui/LY/2021, and A/Duck/Gaoyou/4D1/1/2021-formed a closely related subclade that grouped with other avian H3N2 viruses from China and other parts of Asia, representing a distinct avian evolutionary branch. The canine-derived viruses formed a separate clade distinct from the human and the avian lineage. The strain isolated in this study, A/Canine/Nanjing/CnNj01-2025, was positioned centrally in this cluster and closely grouped with other canine H3N2 viruses circulating in China, the United States, South Korea, and Thailand. Phylogenetic classification placed A/Canine/Nanjing/CnNj01-2025 within the clade 5.1 lineage of CIV-H3N2. The topology indicated that the canine clade diverged from within the avian lineage, with the canine branch embedded in the broader avian evolutionary background. In contrast, the human and avian clades were separated by long branch lengths, indicating substantial evolutionary divergence between human- and avian-derived H3N2 viruses.

### 3.3. Amino Acid Comparison of Major Antigenic Sites on the HA Protein of H3N2 Viruses

**Figure 2:**
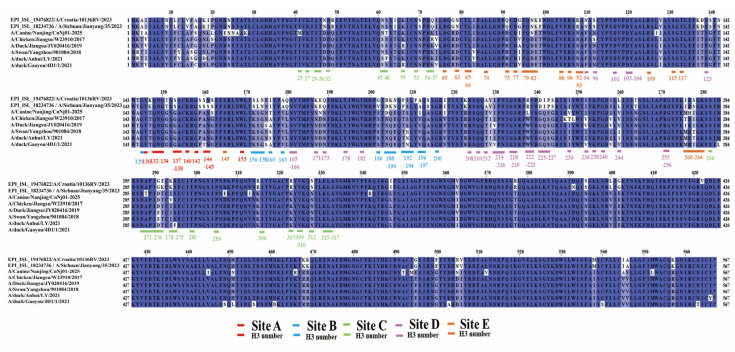
Multiple sequence alignment of five major antigenic sites (A–E) in HA protein of H3N2 influenza viruses. The alignment displays amino acid sequences of the five major antigenic sites (A–E) in HA protein. Sequences were aligned using MAFFT algorithm in PhyloSuite platform. The five antigenic sites are marked with color–coded underlines: Site A (red), Site B (blue), Site C (green), Site D (purple), and Site E (orange). Key positions are labeled with dual numbering format (HA position/H3 position). Amino acids are color–coded by chemical properties. Strains shown: EPI_ISL_18760734 and EPI_ISL_18751261 (human isolates), A/Canine/Nanjing/CnNj01–2025 (canine isolate), A/Chicken/Jiangsu/W23910/2017, A/Duck/Jiangsu/JY020416/2019, A/Swan/Yangzhou/901084/2018, A/duck/Anhui/LY/2021, and A/duck/Gaoyou/4D1/1/2021 (avian isolates). The asterisk (*) denotes a stop codon in the amino acid sequence. The colored backgrounds indicate amino acids located within different antigenic sites: Site A (red), Site B (blue), Site C (green), Site D (purple), and Site E (orange).

Amino acid sequence alignment of the five major antigenic sites (Sites A–E) on the HA protein revealed differences between the canine isolate A/Canine/Nanjing/CnNj01-2025 and both human and avian H3N2 viruses (Figure 2, Table 2). Within antigenic site B, the canine strain carried a substitution I176/160T, which has been reported to influence the spatial conformation of residue 158 in certain avian influenza subtypes. At residue 204/188, the canine strain encoded aspartic acid (D), consistent with human strains A/Croatia/l0136RV/2023 and A/Sichuan/Jianyang/35/2023, whereas all avian strains encoded asparagine (N), indicating a D204/188N substitution in avian strains. This residue lies adjacent to the receptor-binding site at 205/189. The A212/196I mutation in the canine strain also represents a potential marker of antigenic drift. In antigenic site C, the canine strain encoded asparagine(N61/45), identical to human and some avian strains. The most pronounced differences were observed in antigenic Sites D and E. At residue 237/222, the canine strain encoded leucine (L), whereas human strains encoded arginine (R), and avian strains encoded tryptophan (W), corresponding to L237/222R in human viruses and L237/222W in avian viruses. At residue 97/81, both the canine and human strains encoded asparagine (N), while all avian strains retained aspartic acid (D), corresponding to the D97/81N substitution.

### 3.4. Analysis of N-Linked Glycosylation Sites on the HA Protein of H3N2 Viruses

Prediction of N-linked glycosylation sites on the HA proteins of eight H3N2 influenza virus strains revealed distinct host-associated glycosylation patterns. The two human strains, A/Croatia/l0136RV/2023 and A/Sichuan/Jianyang/35/2023, exhibited 12 glycosylated sites among 15 predicted motifs: 24, 38, 54, 61, 79, 110, 142, 149, 181, 262, 301, and 499, while residues 18, 22, and 97 were not glycosylated. The canine strain A/Canine/Nanjing/CnNj01-2025 exhibited a markedly different profile, with only seven glycosylated residues: 38, 54, 61, 97, 181, 301, and 499. Notably, glycosylation at residue 97 was uniquely present in the canine strain and absent from both human viruses. The canine strain lacked glycosylation at residues 24, 79, 110, 142, 149, and 262, all of which were glycosylated in human strains (Table 3). The five avian strains—A/Chicken/Jiangsu/W23910/2017, A/Duck/Jiangsu/JY020416/2019, A/Swan/Yangzhou/901084/2018, A/Duck/Anhui/LY/2021, and A/Duck/Gaoyou/4D1/1/2021—showed highly conserved glycosylation patterns. Among them, only A/Duck/Jiangsu/JY020416/2019 was glycosylated at residue 18, while the others were not. All avian strains were glycosylated at residue 22, while lacking glycosylation at residues 24, 79, 97, 110, 142, 149, and 262. This reflects a glycosylation profile distinct from those observed in human and canine viruses. Of particular interest, glycosylation at residue 97 exhibited clear host-specificity in the canine strain.

**Figure 3:**
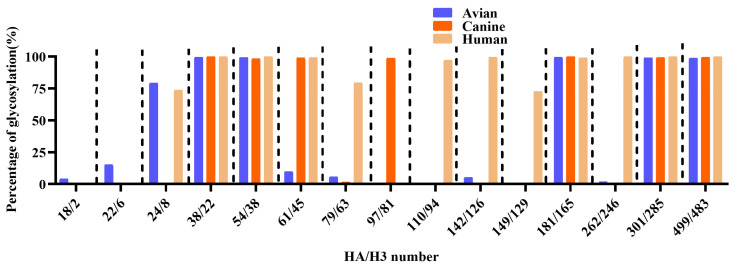
Comparative analysis of potential N-linked glycosylation sites in HA protein among avian, canine, and human H3N2 influenza viruses.

A comprehensive analysis of 685 avian-derived, 349 canine-derived, and 19,527 human-derived H3N2 HA sequences revealed both conserved and variable glycosylation patterns across host species. Glycosylation at HA38/22, 54/38, 181/165, 301/285, and 499/483 was consistently detected at high frequencies across all groups (>98%), indicating strong evolutionary conservation at these sites. In contrast, HA18/2 and HA22/6 exhibited moderate glycosylation frequencies in avian viruses (4.06% and 15.04%, respectively), while they were nearly absent in both canine and human strains (≤0.05%). The HA24/8 site showed high glycosylation prevalence in avian (79.10%) and human strains (73.73%), while detection in canine strains was markedly reduced (0.58%). Glycosylation at HA79/63 and HA110/94 was rarely observed in avian and canine viruses, while it was prominent in human strains, indicating human-specific acquisition of these modifications during host adaptation. In contrast, glycosylation at HA97/81 was detected exclusively in canine strains, consistent with a canine-specific adaptation event following avian-to-canine transmission. Glycosylation at HA142/126 and H149/A129 remained extremely rare in avian strains and was absent in all canine viruses. HA262/246 was infrequently glycosylated in avian (1.79%) and canine (0.57%) strains, while it appeared highly glycosylated in human strains (99.90%) (Figure 3 and Supplementary Table S2). Collectively, these findings demonstrate that several HA/H3 glycosylation sites undergo dynamic remodeling, reflecting adaptation and potential antigenic drift in different host species.

### 3.5. Structural Analysis of Glycosylation Patterns and Key Antigen Residues on the HA Trimer

**Figure 4:**
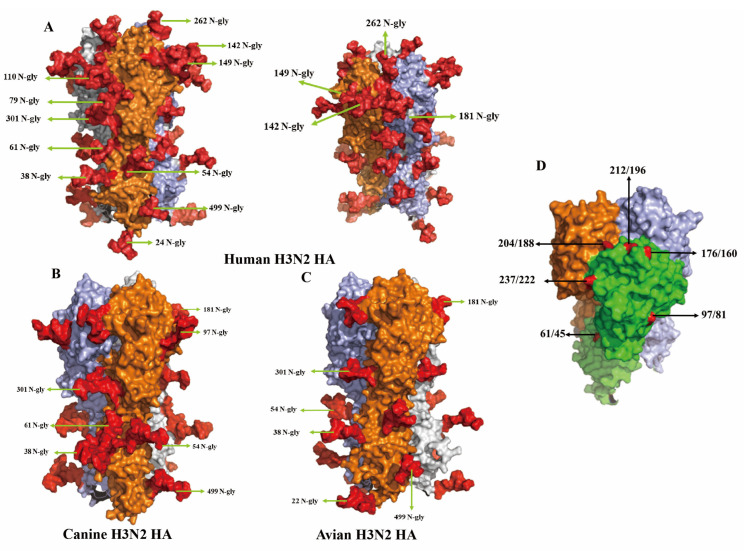
Three-dimensional structure comparison of N-glycosylation sites (**A**–**C**) and antigen sites (**D**) on HA proteins from human, canine, and avian H3N2 influenza viruses. (**A**) Human H3N2 HA protein trimeric structure (A/Sichuan/Jianyang/35/2023). Three HA monomers are shown in different colors (orange, light blue, and gray) to distinguish individual subunits. N-glycosylation sites are labeled with position numbers and “N-gly”. The red area represents the glycosylated sites (**B**) Canine H3N2 HA protein trimeric structure (A/Canine/Nanjing/CnNj01-2025). (**C**) Avian H3N2 HA protein trimeric structure (A/Duck/Jiangsu/JY020416/2019). (**D**) Side view of HA trimer showing structural domains and key amino acid positions. The globular head domain (green) and stem region (light green) are color-coded. Key antigenic site positions are labeled in dual numbering format (HA number/H3 number): 176/160, 204/188, 212/196, 237/222, 61/45, and 97/81.

Three-dimensional structural modeling of the HA protein revealed marked differences in glycosylation patterns among H3N2 viruses in different hosts. Figure 4A presents the trimeric HA structure of the human H3N2 strain A/Sichuan/Jianyang/35/2023, with each monomer rendered in a distinct color. This strain contains 12 N-linked glycosylation sites, primarily located on the globular head domain, including residues 24, 38, 54, 61, 79, 110, 142, 149, 181, 262, 301, and 499. These glycans, highlighted in red, form a dense glycan shield across the surface of the HA1 subunit. In contrast, the canine strain A/Canine/Nanjing/CnNj01-2025 possesses a substantially reduced glycosylation profile, retaining only seven sites (38, 54, 61, 97, 181, 301, 499). Notably, it uniquely acquires glycosylation at residue 97, located within antigenic site E (Figure 4B). This glycosylation is specific to the canine strain and absent in both human and avian viruses. The avian strain A/Duck/Jiangsu/JY020416/2019 displayed a third, distinct glycosylation pattern, characterized by a glycan at residue 22 and the absence of glycosylation at residue 61 (Figure 4C). Figure 4D provides a side view of the HA trimer, emphasizing the spatial distribution of key antigenic residues. Residues 176/160, 204/188, and 212/196 (site B) are positioned on the lateral and apical surfaces of the globular head, while residue 61/45 (site C) is situated on the lower portion of the head domain. Residue 237/222 (site D) is exposed on the outer face, and residue 97/81 (site E) occupies a prominent location on the head surface, underscoring its potential relevance in host-specific glycosylation and antigenic variation.

### 3.6. Genome-Wide Analysis of Functional Sites in the Canine H3N2 Isolate

Functional site analysis across seven gene segments identified multiple amino acid substitutions associated with mammalian adaptation in the canine H3N2 strain (Table 4). Key mammalian adaptation markers shared with the human strain A/Sichuan/Jianyang/35/2023 included PB2-292T and PB2-590S, both of which have been implicated in enhanced polymerase activity in mammalian hosts. In contrast, the canine strain retained PB2-627E, which is a residue typical of avian strains, whereas the human strain encoded the mammalian-adaptive PB2-627K substitution. Additional mutations with potential roles in mammalian adaptation were observed in PA, M1, and NS1 proteins. Notably, glycosylation site analysis showed that the canine strain retained an avian-like NA glycosylation pattern, lacking the N367 site present in the human strain. Detailed amino acid comparisons and functional annotations are summarized in Table 4.

### 3.7. HI Assay Results

The HI assay results demonstrated that the human-derived strain A/Sichuan/Jianyang/35/2023 exhibited the highest reactivity with its homologous antiserum, with an HI titer of 7 log_2_. The canine-derived strain A/Canine/Nanjing/CnNj01-2025 showed moderate cross-reactivity, yielding an HI titer of 3 log_2_. In contrast, all five avian-derived strains A/Chicken/Jiangsu/W23910/2017, A/Duck/Jiangsu/JY020416/2019, A/Swan/Yangzhou/901084/2018, A/Duck/Anhui/LY/2021, and A/Duck/Gaoyou/4D1/1/2021 exhibited HI titers of 0, indicating no detectable cross-reactivity with the human antiserum. These results reflect substantial antigenic divergence among canine-, avian-, and human-derived H3N2 influenza viruses.

### 3.8. Receptor-Binding Properties of the Canine H3N2 Isolate

**Figure 5:**
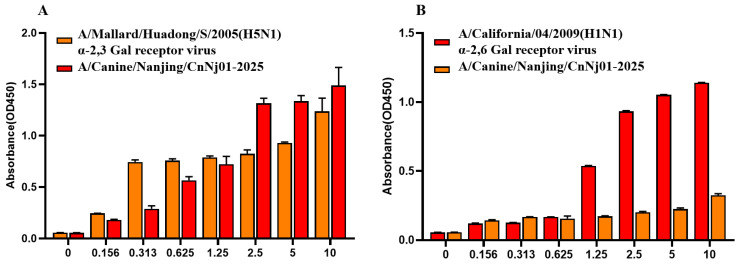
Receptor binding specificity analysis of canine H3N2 influenza virus. (**A**) Binding to avian-type α-2,3-sialylglycopolymer receptor. (**B**) Binding to human-type α-2,6-sialylglycopolymer receptor. A/Mallard/Huadong/S/2005 (H5N1) and A/California/04/2009 (H1N1) were used as control viruses. Solid-phase binding assay was performed with serially diluted glycopolymers (0–10 μg/mL). Error bars represent standard deviations from three independent experiments.

To assess the sialic acid receptor-binding properties of the canine H3N2 isolate, a solid-phase binding assay was performed. Human influenza virus A/California/04/2009 (H1N1), which preferentially binds to sialic acid (SA) α-2,6-galactose (Gal) receptors, and avian influenza virus A/Mallard/Huadong/S/2005 (H5N1), which specifically binds to SA α-2,3-Gal receptors, were included as controls. As illustrated in Figure 5A, the canine strain A/Canine/Nanjing/CnNj01-2025 exhibited strong binding affinity toward α-2,3-linked sialic acid receptors. The binding curve displayed a clear concentration-dependent increase, and at the highest tested concentration (10 μg/mL), binding intensity was comparable to that of A/Mallard/Huadong/S/2005. As shown in Figure 5B, the canine isolate also demonstrated detectability while weaker binding to α-2,6-linked sialic acid receptors, with overall binding levels substantially lower than those of A/California/04/2009 (H1N1). These findings suggest that while the canine H3N2 virus retains a strong preference for avian-type α-2,3 receptors, it has acquired limited binding capacity for human-type α-2,6 receptors.

## 4. Discussion

Viruses belonging to Clade 5 and Clade 5.1, which emerged after 2016, accumulated multiple key mutations in the HA gene, including HA-G146S, HA-N188D. Phylogenetic analysis revealed that our newly isolated strain, A/Canine/Nanjing/CnNj01-2025, clusters within the Clade 5.1 lineage. Consistent with previously reported genetic features of this clade, this isolation carries key HA substitutions such as G146S and N188D. These mutations collectively conferred dual receptor-binding capability-recognizing both α2,3- and α2,6-linked sialic acid receptors-and significantly increased acid and thermal stability [9]. The canine strain A/Canine/Nanjing/CnNj01-2025 carries several HA mutations previously reported to contribute to canine host adaptation. The substitution I176/160T lies outside the seven canonical antigenic sites (145, 155, 156, 158, 159, 189, 193) defined by Koel et al. [25]. Although not located in major antigenic regions, Lewis et al. showed that substitutions within the 158–160 stretch can shift antigenic distance by 3–8 units [42].

Glycosylation-site analysis further differentiated the canine-derived strain from its human-derived counterpart. NetNGlyc prediction indicated that the canine HA sequence possesses seven potential N-linked glycosylation sites (38, 54, 61, 97, 181, 301, 499), whereas the contemporary human H3N2 strain contains twelve (24, 38, 54, 61, 79, 110, 142, 149, 181, 262, 301, 499). A critical distinction lies at residue 97/81: the canine virus retains a glycosylation motif at this site, whereas current human H3N2 viruses have lost this modification. Retrospective studies revealed that the 1968 H3N2 pandemic virus initially exhibited two glycosylation sites, N81 and N165, in the HA globular head [43]. Kobayashi & Suzuki reported that the glycosylation motif at site 81 was lost in human viruses by 1974, a loss closely associated with the gain of glycosylation at site 63, the two motifs covering overlapping antigenic epitopes [44]. Bradley et al. similarly noted the presence of glycosylation at site 81 in early pandemic strains such as A/Aichi/2/68 with absence in human H3N2 sequences after 1974 [45]. In the present study, the canine virus retains glycosylation at residue 97/81, resulting in a glycosylation pattern that resembles early human H3N2 viruses (1968–1974) rather than that of modern seasonal strains. Research by Das et al. showed that N-linked glycans can form glycan shields that mask antigenic epitopes and reduce antibody binding efficiency [46]. The glycosylation retained at 97/81 in the canine virus may influence the exposure of adjacent antigenic sites, whereas human viruses appear to have compensated for the loss at residue 81 by acquiring glycosylation at sites 63, 126, and 129, establishing a distinct glycan-shield configuration. Notably, the glycosylation site at 97/81, which is conserved in canine influenza viruses while absent in most avian and human strains, corresponds to a unique antigenic region. This site may serve as a host-specific feature contributing to differential antibody recognition and shaping the antigenic profiles of CIV-H3N2.

In addition to the head-domain dynamics, the HA stem region demonstrates different evolutionary patterns at the 61/45 glycosylation sites during the avian-to-mammalian transition. Site 61/45 exhibits an acquisition trajectory: this site remains predominantly unglycosylated in avian strains yet achieves near-complete glycosylation occupancy (approaching 100%) in both canine and human H3N2 viruses. This convergent glycosylation gain across independent mammalian lineages suggests that modification at 61/45 may contribute to successful interspecies adaptation. Kobayashi & Suzuki demonstrated through natural selection analysis that glycosylation at site 61/45 masks stem epitope C, reduces the dN/dS ratio from >1 to <1 at adjacent antigenic residues, and thereby attenuates antibody-mediated neutralization [44].

Ushirogawa et al. reported that since the emergence of human H3N2 influenza in 1968, the number of N-linked glycosylation sites on HA has increased gradually from two to approximately twelve due to antigenic drift [47]. In contrast, the canine strain examined herein contains only seven glycosylation motifs. The comparatively limited glycosylation profile may reflect the shorter period of canine circulation and correspondingly lower cumulative immune-selection pressure. Wu & Wilson noted that host-specific immune systems impose distinct selective pressures on influenza viruses, thereby shaping different antigenic-evolution trajectories across hosts [48]. Consistent with this, the canine virus retains glycosylation at 97/81 yet lacks several glycosylation sites commonly acquired in human H3N2 viruses at 79/63, 110/94, 142/126, 149/129, and 262/246, potentially causing additional exposure of antigenic epitopes. Although Lewis et al. found that glycosylation plays a modulatory role in antigenic evolution of swine H3N2 viruses, they concluded that major antigenic shifts are principally driven by specific amino-acid substitutions [42]. In this context, the combination of amino-acid substitutions I176/160T, N204/188D, A212/196I, and W237/222L together with retention of the glycosylation motif at 97/81 and the absence of several human-virus-specific glycosylation sites support the inference that the canine H3N2 virus has acquired antigenic characteristics clearly distinct from those of current human strains. These multiple amino-acid changes likely contribute to substantial antigenic differentiation. Nevertheless, divergence is not absolute. The coexistence of partially convergent antigenic residues and a unique glycosylation profile may explain the moderate serological cross-reactivity observed in hemagglutination-inhibition assays, suggesting that while canine H3N2 viruses are evolving antigenically in a direction partially aligned with human strains, they retain distinctive molecular signatures.

The integration of hemagglutination inhibition assays, antigenic site mapping, glycosylation profiling, and receptor-binding quantification reveals a coordinated evolutionary strategy by which canine H3N2 viruses balance the selective pressures of immune evasion and host adaptation. The observed 16-fold reduction in HI titer between the canine strain and the human reference strain A/Sichuan/Jianyang/35/2023, together with a complete lack of cross-reactivity with avian strains, reflects the cumulative impact of multiple amino acid substitutions operating synergistically rather than additively across the HA structure. Recent structural analyses demonstrate that antigenic drift in H3N2 viruses emerges through epistatic interactions among distant residues, where mutations outside canonical antigenic sites modulate epitope accessibility through long-range conformational effects [49]. The five key substitutions identified in the canine strain, namely D97/81N, A176/160T, N204/188D, V212/196I, and W237/222L, span antigenic sites B, D, and E, creating a novel antigenic surface topology distinct from both avian progenitor viruses and contemporary human strains. Among these, the D97/81N mutation introduces a unique N-glycosylation site observed exclusively in canine H3N2 lineages, positioning a carbohydrate shield within antigenic site E. However, recent deep mutational scanning studies reveal that glycosylation at individual sites rarely confers antigenic escape in isolation; instead, glycans can synergistically with surrounding amino acid substitutions to reshape antibody binding trajectories [50]. The canine strain exhibits an intermediate glycosylation profile, retaining seven N-glycans compared with twelve human strains, suggesting a shorter evolutionary history in the canine host and correspondingly lower cumulative immune-selection pressure. This pattern aligns with historical observation that human H3N2 viruses experienced rapid glycosylation site acquisition between 1997 and 2002, with each additional glycosylation site contributing not only to epitope masking but also to modulation of receptor accessibility and viral fitness under host immune pressure [51]. The receptor-binding phenotype, characterized by strong avian α-2,3 sialic-acid affinity with modest human α-2,6 sialic-acid recognition, cannot be fully explained by mutations within the canonical receptor-binding pocket itself, which remain largely conserved. Instead, the W237/222L substitution exemplifies how peripheral antigenic site mutations exert allosteric control over receptor specificity, with position 222 lying at the base of the 220-loop where conformational changes propagate to alter receptor-binding geometry without direct sialic acid contact [28,52]. The N204/188D substitution adjacent to the critical receptor-binding residue 205/189 may also affect binding through electrostatic modulation of sialic acid coordination angles, an increasingly recognized mechanism in host range expansion in zoonotic influenza viruses [53]. Importantly, the coexistence of retained avian-type binding with limited α-2,6 recognition indicates incomplete receptor adaptation. The discordance between partial antigenic convergence with human strains, reflected by measurable HI cross-reactivity, and incomplete receptor adaptation, reflected in modest α-2,6 binding, suggests that antigenic evolution proceeds more rapidly than functional optimization during host transitions. This may create a temporary window where viruses are partially recognizable by human antibodies yet remain functionally constrained from efficient human infection [1,9]. This evolutionary asymmetry likely reflects differing selective pressures: antigenic sites under direct antibody-mediated selection evolve rapidly through single amino acid changes with immediate fitness benefits, whereas receptor-binding optimization requires coordinated mutations across multiple residues to maintain the delicate balance between binding affinity, specificity, and HA stability [52,54]. The integration of these multidimensional data thus reveals that canine H3N2 occupies a distinct adaptive landscape characterized by antigenic divergence from avian ancestors, partial serological overlap with human strains, and incremental receptor adaptation. This molecular signature is consistent with ongoing mammalian host adaptation but insufficient human-specific optimization to support canine-to-human transmission.

Analysis of internal gene segments revealed additional molecular signatures distinguishing avian and mammalian H3N2 lineages. In the PB2 polymerase subunit, the canine strain harbors mutations I292T and G590S. Soh et al. conducted a comprehensive deep mutational scanning of the PB2 gene and identified I292T as one of the mutations that improve polymerase activity in human cells [55]. Mehle and Doudna demonstrated that the G590S/Q591R polymorphism enhances viral polymerase function in mammalian cells and contributes to adaptation in human hosts [32]. Within the PA polymerase subunit, the C241Y substitution represents a critical host-range determinant. Yamaji et al. demonstrated through reverse genetics that PA-C241Y significantly enhances viral replication and pathogenicity in mammalian models, identifying this mutation as one of five key mammalian-adaptive substitutions in the PA protein of highly pathogenic avian H5N1 influenza viruses [35]. The PB1 polymerase subunit exhibits the substitution G216S in the canine strain. Lin et al. characterized this position as a mammalian signature site, demonstrating that PB1 216G is predominantly present in human influenza A viruses and affects viral replication fidelity and virulence in mammalian systems [34]. Collectively, these internal gene modifications complement the surface glycoprotein adaptations described above, establishing a comprehensive molecular framework that facilitates H3N2 virus colonization of mammalian hosts through optimization of the polymerase complex for efficient replication under mammalian cellular conditions.

The findings of this study indicate that although the canine-origin H3N2 virus exhibits significant molecular differences from human and avian strains, its observed antigenic drift suggests that the virus continues to undergo evolutionary changes. To achieve effective surveillance of canine influenza viruses, a systematic and sustained epidemiological monitoring framework should be established. Currently, global influenza surveillance primarily relies on the World Health Organization’s Global Influenza Surveillance and Response System (GISRS), whereas surveillance for canine influenza remains relatively limited, lacking standardization and cross-sectoral coordination. Future monitoring of canine influenza should emphasize integration with existing human and avian influenza surveillance systems by establishing a joint network encompassing veterinary institutions and public health laboratories. For newly isolated canine H3N2 strains, hemagglutination inhibition (HI) assays should be routinely performed to compare antigenic characteristics with human vaccine and circulating strains, thereby tracking trends in antigenic drift. In parallel, phylogenetic analyses and receptor-binding assays should be conducted to assess host adaptation dynamics. Strains exhibiting significant changes in receptor-binding specificity or antigenic properties should be further evaluated in mammalian models to determine their pathogenicity and transmission potential.

This study isolated a canine H3N2 virus A/Canine/Nanjing/CnNj01-2025, which belongs to an avian-origin evolutionary lineage. Comparative analysis of HA antigenic sites identified key mutations in the canine strain at residues 176/160, 204/188, 212/196, and 237/222, which, according to serological assays, have led to the re-establishment of cross-reactivity between canine and human H3N2 viruses. Glycosylation analysis showed that the canine virus retained glycosylation at residue 97/81, distinguishing it from contemporary human and avian strains. Receptor-binding assays demonstrated that the canine strain has acquired low-level binding affinity for α-2,6-linked sialic acid receptors. These findings highlight the importance of continuous epidemiological and antigenic surveillance of canine influenza viruses, with particular emphasis on comparative monitoring with human influenza strains.

## Figures and Tables

**Table 1:** Influenza virus strains used for comparative analysis in this study.

Strain Name	Abbreviation	Host	Collection Date	Geographic Origin	GenBank/GISAID Accession No.
A/Croatia/l0136RV/2023	H1	Human	2023	Croatia	EPI_ISL_19476822
A/Sichuan/Jianyang/35/2023	H2	Human	2023	Sichuan, China	EPI2735848-EPI2735855
A/Canine/Nanjing/CnNj01-2025	C1	Canine	March 2025	Nanjing, Jiangsu, China	PX474832-PX474839
A/Chicken/Jiangsu/W23910/2017	A1	Avian (Chicken)	2017	Jiangsu, China	PV124747-PV124754
A/Duck/Jiangsu/JY020416/2019	A2	Avian (Duck)	2019	Jiangsu, China	PV124763-PV124770
A/Swan/Yangzhou/901084/2018	A3	Avian (Swan)	2018	Yangzhou, Jiangsu, China	PV124739-PV124746
A/Duck/Anhui/LY/2021	A4	Avian (Duck)	2021	Anhui, China	PV124795-PV124802
A/Duck/Gaoyou/4D1/1/2021	A5	Avian (Duck)	2021	Gaoyou, Jiangsu, China	PV124803-PV124810

**Table 2:** Key amino acid variations at HA antigenic sites in canine H3N2.

	HA/H3 Number	H1	H2	C1	A1	A2	A3	A4	A5	Phenotype
Site B	176/160	I	I	T	A	A	S	S	A	Affected the amino acid orientation at residue 158 [22,23]
	204/188	D	D	D	N	N	N	N	N	Close to key antigenic site and receptor binding site 205/189 [24,25]
	212/196	A	A	I	V	V	V	V	V	Important for antigenic drift [26]
Site C	61/45	N	N	N	S	S	N	S	S	Site C [27]
Site D	237/222	R	R	L	W	W	W	W	W	The mutation at site 222 directly impacts viral fitness and antigenicity [28]
Site E	97/81	N	N	N	D	D	D	D	D	D81N influences antigenic drift and generates glycosylation only in CIV [28]

Note: A/Croatia/l0136RV/2023(H1), A/Sichuan/Jianyang/35/2023(/H2), A/Canine/Nanjing/CnNj01-2025(C1), A/Chicken/Jiangsu/W23910/2017(A1), A/Duck/Jiangsu/JY020416/2019(A2), A/Swan/Yangzhou/901084/2018(A3), A/duck/Anhui/LY/2021(A4), A/duck/Gaoyou/4D1/1/2021(A5). Phenotype column describes the functional significance of each substitution based on published literature. Site B, C, D, and E refer to the five major antigenic sites on H3 HA.

**Table 3:** N-glycosylation site prediction of HA protein in H3N2 influenza viruses.

HA/H3 Number	18/ 2	22/ 6	24/ 8	38/ 22	54/ 38	61/ 45	79/ 63	97/ 81	110 /94	142 /126	149 /129	181 /165	262 /246	301 /285	499 /483
H1	−	−	+	+	+	+	+	−	+	+	+	+	+	+	+
H2	−	−	+	+	+	+	+	−	+	+	+	+	+	+	+
C1	−	−	−	+	+	+	−	+	−	−	−	+	−	+	+
A1	−	+	−	+	+	−	−	−	−	−	−	+	−	+	+
A2	+	+	−	+	+	−	−	−	−	−	−	+	−	+	+
A3	−	+	−	+	+	+	−	−	−	−	−	+	−	+	+
A4	−	+	−	+	+	−	−	−	−	−	−	+	−	+	+
A5	−	+	−	+	+	−	−	−	−	−	−	+	−	+	+

Note: A/Croatia/l0136RV/2023(H1), A/Sichuan/Jianyang/35/2023(/H2), A/Canine/Nanjing/CnNj01-2025(C1), A/Chicken/Jiangsu/W23910/2017(A1), A/Duck/Jiangsu/JY020416/2019(A2), A/Swan/Yangzhou/901084/2018(A3), A/duck/Anhui/LY/2021(A4), A/duck/Gaoyou/4D1/1/2021(A5). “+” indicates the presence of N-glycosylation site; “−” indicates the absence of N-glycosylation site. Amino acid positions on HA are presented in dual format (HA/H3 numbering).

**Table 4:** The major amino acids of strains in this study may affect functions.

	Residues	H2	C1	A1	A2	A3	A4	A5	Phenotype
PB2	147	I	T	I	I	I	I	I	I147T is critical for high virulence in mammals [29,30]
	292	T	T	I	I	I	I	I	I292T increases viral polymerase in H9N2 [31]
	590	S	S	G	G	G	G	G	G590S is adaptation marker in H1N1 [32]
	627	K	E	E	E	E	E	E	E627K is determinant of host range [33]
PB1	216	G	N	S	S	S	S	N	G216S shows higher virulence in mammal [34]
PA	241	C	Y	C	C	C	C	C	C241Y and other sites markedly enhanced virus growth in the lung tissue of mice [35]
	383	N	D	D	D	D	D	D	N383D substitutions contribute to mammalian adaptation [36]
	573	V	I	I	I	I	I	I	I573V and other sites markedly enhanced virus growth in the lung tissue of mice [35]
NP	313	Y	F	F	F	F	F	F	
	357	K	Q	Q	Q	Q	Q	Q	Q357K determines the virulence phenotype in mice [37]
NA	367–369	NET	SKD	SKD	SKD	SKD	SKD	SKD	N367 is an N-linked glycosylation site [38]
	402–403	DR	NR	NR	NR	NR	NR	NR	N402 is an N-linked glycosylation site [38]
M1	15	V	I	V	I	V	V	V	The V15I mutation in H5N1 virus is associated with enhanced virulence [39]
NS1	67	K	W	R	R	R	R	R	R67W is related to viral host adaptability and virulence [40]
	75	E	K	E	E	E	E	E	Participates in the formation of type I β-turn [41]

Note: The strain A/Croatia/l0136RV/2023 (H1) was included in the analysis; however, its internal gene sequences were not available, A/Sichuan/Jianyang/35/2023(/H2), A/Canine/Nanjing/CnNj01-2025(C1), A/Chicken/Jiangsu/W23910/2017(A1), A/Duck/Jiangsu/JY020416/2019(A2), A/Swan/Yangzhou/901084/2018(A3), A/duck/Anhui/LY/2021(A4), A/duck/Gaoyou/4D1/1/2021(A5). Amino acid sites are numbered according to the respective protein sequences. Phenotype column describes the functional implications based on published literature.

## Data Availability

The original contributions presented in this study are included in the article and Supplementary Material. Further inquiries can be directed to the corresponding authors. The genomic sequences of A/Canine/Nanjing/CnNj01-2025 virus are available in GenBank under the accession numbers PX474832-PX474839.

## Supplementary Materials

The following supporting information can be downloaded at https://www.mdpi.com/article/10.3390/vetsci13010032/s1. Supplementary Table S1. H3N2 influenza virus strains used for phylogenetic analysis; Supplementary Table S2. Glycosylation site analysis of influenza virus strains.
