# Supplementary material for: Molecular Characterization of an H3N2 Canine Influenza Virus Isolated from a Dog in Jiangsu, China, in 2025
Source: Vet Sci. 2025 Dec 29;13(1):32. doi: 10.3390/vetsci13010032 (PMC12846395; doi:10.3390/vetsci13010032)
Supplement: Supplementary file 1 [file vetsci-13-00032-s001.zip › Supplementary Table S1.pdf]

Molecular Characteristics of an H3N2 Canine Influenza Virus isolated from a dog in Jiangsu, China in 2025

Jingwen Peng[1,2], Xinyu Miao[3], Xinyi Zhang[1], Zifan Li[3], Yiling Wang[3], Guofang Liu[4], Lei Na[4], Nuo Xu[4]*, Daxin Peng[3]*

1 Department of Veterinary Clinical Sciences, College of Veterinary Medicine, Nanjing Agricultural University

2 Nanjing Agricultural University Veterinary Teaching Hospital

3 College of Veterinary Medicine, Yangzhou University

4 College of Animal Husbandry and Veterinary Medicine, Jiangsu Vocational College of Agriculture and Forestry

*Correspondence: E-mail address: Nuo Xu; E-mail address: nuoxu@jsafc.edu.cn. Daxin Peng: pengdx@yzu.edu.cn.

Table S1 H3N2 influenza virus strains used for phylogenetic analysis

Accession number	Viral name
EPI_ISL_18234736	A_Sichuan_Jianyang_35_2023
EPI_ISL_19476822	A_Croatia_10136RV_2023__24_160_
EPI_ISL_107711	A_Fujian_411_2002
EPI_ISL_156425	A_Hangzhou_A812_2013
EPI_ISL_157612	A_Guangdong_ST798_2008
EPI_ISL_18651017	A_shandongrencheng_1417_2019_H3_
EPI_ISL_18681133	A_Yunnan_Linxiang_11036_2017
EPI_ISL_18681233	A_Yunnan_Mengzi_1866_2019
EPI_ISL_18681264	A_Yunnan_Linxiang_130_2021
EPI_ISL_18681270	A_Yunnan_Wenshan_1213_2021
EPI_ISL_18857207	A_Henan_Shihe_1977_2023
EPI_ISL_18862303	A_Shanghai_Fengxian_1912_2023
EPI_ISL_18909438	A_WuhanQiaokou_230380H3_2023
EPI_ISL_19150406	A_Shanxi_Taiyuan_131_2023
EPI_ISL_19460388	A_Shanghai_LW1587C2_2010
EPI_ISL_19460834	A_Shanghai_CM1244C2_2018
EPI_ISL_19460847	A_Shanghai_CM1694C1_2017
EPI_ISL_19460924	A_Shanghai_JD1538C1_2010
EPI_ISL_19461013	A_Shanghai_HK1613C2_2017
EPI_ISL_20169972	A_Jilin_Tiexi_128_2025
EPI_ISL_20180482	A_Xinjiang_Changji_1952_2025
EPI_ISL_20180484	A_Chongqing_Wanzhou_1224_2025
EPI_ISL_20180487	A_Jilin_Ningjiang_1204_2025
EPI_ISL_29271	A_Guangdong_05_2005
EPI_ISL_29273	A_Guangdong_09_2005
EPI_ISL_393365	A_China_51045_2013
EPI_ISL_393412	A_China_67066_2015
EPI_ISL_528885	A_Beijing_Miyun_51_2019
PX474832	A/Canine/Nanjing/CnNj01-2025_H3N2_HA
EPI_ISL_127502	A_canine_Korea_01_2007_HA
EPI_ISL_129987	A_canine_Korea_KRIBB01_2011
EPI_ISL_137602	A_canine_Thailand_CU_DC5299_2012_HA
EPI_ISL_138997	A_canine_Korea_CY009_2010_HA
EPI_ISL_14874671	A_canine_Singapore_SG_NParks_CIV_M4705_2018
EPI_ISL_14874741	A_canine_Singapore_SG_NParks_CIV_M10305_2018
EPI_ISL_151145	A_canine_Guangdong_12_2012
EPI_ISL_16189	A_canine_Korea_GCVP01_2007_HA
EPI_ISL_170565	A_canine_Korea_S1_2012_11_05_2012_HA
EPI_ISL_17277335	A_canine_China_15027_2019
EPI_ISL_17805983	A_dog_Indiana_M16_09378_32_1_2016
EPI_ISL_18690796	A_canine_Beijing_CAU_118_2018

EPI_ISL_18750729	A_canine_California_CVM_3466381_2021
EPI_ISL_18750730	A_canine_Florida_CVM_B1_2021
EPI_ISL_18750734	A_canine_Pennsylvania_CVM_985419_2023
EPI_ISL_18751261	A_canine_West_Virginia_CVM_120508_1_2023
EPI_ISL_18752205	A_canine_Alabama_CVM_194456_1_2022
EPI_ISL_18752209	A_canine_Texas_CVM_042018_3_2023
EPI_ISL_18961353	A_canine_Heilongjiang_1_2019
EPI_ISL_19438001	A_canine_Jilin_01_2023
EPI_ISL_19890775	A_dog_California_25_011941_001_R_2025
EPI_ISL_19890814	A_dog_California_25_011943_001_R_2025
EPI_ISL_218443	A_canine_Korea_BD_1_2013
EPI_ISL_284693	A_canine_South_Korea_0173915_2015
EPI_ISL_366884	A_canine_China_Shanghai_0315_2019_2019
EPI_ISL_372282	A_Canis_lupus_familiaris_USA_007781_2018
EPI_ISL_379494	A_canine_Xi_an_20170601_157_2017
EPI_ISL_379496	A_canine_Shanghai_20170713_18_2017
EPI_ISL_379505	A_canine_Beijing_20161228_9_2016
EPI_ISL_4031576	A_canine_Guangdong_DY1_2019
EPI_ISL_4061869	A_canine_Illinois_21_015197_001_2021
EPI_ISL_499086	A_canine_Guangdong_3_2018
EPI_ISL_502304	A_canine_Ontario_NCFAD_2018_070_7_2018
EPI_ISL_6785379	A_canine_Henan_L03_2018
EPI_ISL_6795312	A_canine_China_A4_2021
EPI_ISL_80505	A_canine_Guangdong_1_2006
EPI_ISL_80507	A_canine_Guangdong_2_2007
EPI_ISL_93812	A_canine_Jiangsu_06_2010
ON877939	A/canine/Shanghai/159/2017(HA)clade_5
ON877723	A/canine/Guangzhou/1180/2019(HA)clade_5.1
ON877819	A/canine/Hainan/07-9/2019(HA)clade_5.1
PV124750	A_Chicken_Jiangsu_W23910_2017_H3N2_HA
PV124766	A_Duck_Jiangsu_JY020416_2019_H3N2_HA
PV124742	A_Swan_Yangzhou_901084_2018_H3N2_HA
PV124798	A_duck_Anhui_LY_2021_H3N2_HA
PV124806	A_duck_Gaoyou_4D1_1_2021_H3N2_HA
EPI_ISL_133705	A_duck_Jiangsu_26_2004
EPI_ISL_13732934	A_Goose_Guangdong_G630_2019_H3N2
EPI_ISL_13734228	A_Chicken_Sichuan_H157_2020_H3N2
EPI_ISL_167461	A_mallard_Jiangxi_1_4_2010
EPI_ISL_18708429	A_duck_China_GVRI_401D23_2019
EPI_ISL_18708431	A_duck_China_GVRI_403D49_2020
EPI_ISL_18718874	A_duck_China_GVRI_402D22_2019
EPI_ISL_19243986	A_goose_China_318G39_2018
EPI_ISL_19244027	A_chicken_China_284C1_2017
EPI_ISL_19244046	A_duck_China_272D18_2016

EPI_ISL_19733239	A_duck_Anhui_LY_2021
EPI_ISL_19733243	A_duck_Jiangsu_JY020416_2019
EPI_ISL_19818731	A_duck_Chongqing_M3923_2023
EPI_ISL_19818733	A_duck_Fujian_F1219_2022
EPI_ISL_19818739	A_duck_Guangdong_F1014_2022
EPI_ISL_19818741	A_duck_Jiangsu_M21539_2023
EPI_ISL_19818744	A_duck_Jiangsu_M6175_2023
EPI_ISL_19818758	A_duck_Liaoning_F0223_2023
EPI_ISL_212045	A_duck_Anhui_D293_2014
EPI_ISL_217488	A_Bean_Goose_Hubei_chenhu__XVI35_1_2016_H3N2
EPI_ISL_224895	A_duck_Hebei_B1646_2_2011
EPI_ISL_252830	A_chicken_Ganzhou_GZ157_2016
EPI_ISL_282917	A_duck_Jiangshu_YZ916_2016
EPI_ISL_365540	A_duck_Guangxi_S4873_2014
EPI_ISL_365549	A_duck_Guangxi_S3671_2014
EPI_ISL_6780603	A_duck_China_322D22_2018
EPI_ISL_6781438	A_duck_China_Influenza
EPI_ISL_93961	A_duck_Korea_JS53_2004_HA
